# Low-Temperature-Induced Expression of Rice *Ureidoglycolate Amidohydrolase* is Mediated by a C-Repeat/Dehydration-Responsive Element that Specifically Interacts with Rice C-Repeat-Binding Factor 3

**DOI:** 10.3389/fpls.2015.01011

**Published:** 2015-11-13

**Authors:** Juan Li, Rui-Ying Qin, Hao Li, Rong-Fang Xu, Ya-Chun Yang, Da-Hu Ni, Hui Ma, Li Li, Peng-Cheng Wei, Jian-Bo Yang

**Affiliations:** Key Laboratory of Rice Genetic Breeding of Anhui Province, Rice Research Institute, Anhui Academy of Agricultural SciencesHefei, China

**Keywords:** CRT/DRE element, low temperature stress, OsCBF3, transcriptional regulation, *ureidoglycolate amidohydrolase*

## Abstract

Nitrogen recycling and redistribution are important for the environmental stress response of plants. In non-nitrogen-fixing plants, ureide metabolism is crucial to nitrogen recycling from organic sources. Various studies have suggested that the rate-limiting components of ureide metabolism respond to environmental stresses. However, the underlying regulation mechanism is not well understood. In this report, rice *ureidoglycolate amidohydrolase* (*OsUAH*), which is a recently identified enzyme catalyzing the final step of ureide degradation, was identified as low-temperature- (LT) but not abscisic acid- (ABA) regulated. To elucidate the LT regulatory mechanism at the transcriptional level, we isolated and characterized the promoter region of *OsUAH* (P*_OsUAH_*). Series deletions revealed that a minimal region between –522 and –420 relative to the transcriptional start site was sufficient for the cold induction of P*_OsUAH_*. Detailed analyses of this 103-bp fragment indicated that a C-repeat/dehydration-responsive (CRT/DRE) element localized at position –434 was essential for LT-responsive expression. A rice C-repeat-binding factors/DRE-binding proteins 1 (CBFs/DREB1s) subfamily member, OsCBF3, was screened to specifically bind to the CRT/DRE element in the minimal region both in yeast one-hybrid assays and in *in vitro* gel-shift analysis. Moreover, the promoter could be exclusively *trans*-activated by the interaction between the CRT/DRE element and OsCBF3 *in vivo*. These findings may help to elucidate the regulation mechanism of stress-responsive ureide metabolism genes and provide an example of the member-specific manipulation of the CBF/DREB1 subfamily.

## Introduction

Metabolic adjustments alter the physiological and developmental reactions of plant stress adaptation (Bohnert et al., 1995; Bohnert and Sheveleva, 1998). Nitrogen recycling and redistribution are important for the environmental stress response (Nicolas et al., 1985; Tahir and Nakata, 2005; Zhu et al., 2007; Maruyama et al., 2009; Guttieri et al., 2013; Zhang et al., 2014). The redistributed nitrogen is transported among cells or organs of a plant in the form of amino acids or ureides (Schubert, 1986). In most plant species, ureides are the intermediates of the nitrogen recycling from the purine nucleotides for remobilization into amino acids. The functions of ureides have been well documented in the source-to-sink transportation of nitrogen-fixing plants, whereas accumulating evidence has led to the hypothesis that ureides might also participate in stress adaptation. Ureide catabolism is involved in nitrogen recycling from stressed and senescent tissues in drought-treated legumes (Alamillo et al., 2010; Díaz-Leal et al., 2014). Allantoin and allantoate, the primary types of ureides, are accumulated by stresses such as drought, cold, and salinity (Brychkova et al., 2008; Alamillo et al., 2010; Kanani et al., 2010; Yobi et al., 2013). The accumulation of rice grain allantoin is positively correlated with seedling tolerance to low-temperature (LT) stress (Wang et al., 2012). Allantoin has been wildly used as a biomarker of oxidative stress in mammalian cells (Esen et al., 2011; Chung and Benzie, 2013; Fukuhara et al., 2013). Although lacking antioxidant activity *in vitro*, allantoin supplementation could effectively mitigate oxidative damage in plants (Gus’kov et al., 2004; Brychkova et al., 2008). Further evidence has indicated that exogenous allantoin could improve plant resistance to various stresses (Watanabe et al., 2010; Wang et al., 2012). In addition, recent studies in *Arabidopsis* demonstrated that *in vivo*, allantoin is crucial to determine the cellular abscisic acid (ABA) level by activating *de novo* ABA biosynthesis and hydrolysis of the ABA-glucose conjugate (Watanabe et al., 2014a,b).

In plants other than nitrogen-fixing legumes, ureides are converted via the oxidation of purine. Then, ureides are catabolized in a continuous enzymatic reaction to re-assimilate inorganic nitrogen and are finally converted into glycosylate (Werner et al., 2010, 2013; Werner and Witte, 2011). Many ureide generative or degradative enzymes are involved in environmental stresses. For example, in *Arabidopsis*, tomato, sugarcane, and ryegrass, environmental stresses, including LT, drought, and salinity, coincidently upregulated the expression of *xanthine dehydrogenase* (*XDH*), which is rate-limiting enzyme in purine breakdown (Sagi et al., 1998; Nogueira et al., 2003; Hesberg et al., 2004; Yesbergenova et al., 2005). The repression of *Arabidopsis XDH* leads to increasing stress sensitivity (Brychkova et al., 2008; Watanabe et al., 2010). A key enzyme gene of ureides catabolism, *allantoin amidohydrolase* (*ALN*), is upregulated by drought and ABA treatment in some species of common beans (Alamillo et al., 2010; Coleto et al., 2014). Moreover, mutation of the *ALN* gene of *Arabidopsis* greatly enhances the tolerance to water deficit by activating stress-response genes genome-wide (Watanabe et al., 2014b).

Despite several genes of ureide metabolism being associated with stress, the involvement of the remaining components, especially those downstream of allantoate degradation, is largely unknown. More importantly, the regulation mechanisms of stress induction are not well understood. *Ureidoglycolate amidohydrolase* (*UAH*) is a recently identified ureide catabolic enzyme in *Arabidopsis*, rice, and soybeans (Werner et al., 2013) that has the ability *in vitro* to hydrolyze ureidoglycolate into glyoxylate, carbon dioxide, and two molecules of ammonia (Werner et al., 2010). Here, we report the LT-responsive expression of the rice *UAH* gene (*OsUAH, LOC_Os12g40550*), which catalyzes the final step of allantoate degradation (Werner et al., 2010). The molecular mechanism of LT induction of *OsUAH* is investigated. The results obtained here indicate that C-repeat-binding factors/DRE-binding proteins 1 (CBFs/DREB1s) play a critical role in the LT-responsive expression of *OsUAH*. Our results may enhance the understanding of the regulation of stress-involved ureide metabolism genes.

## Materials and Methods

### Plant Materials and Growth Conditions

Rice plants (*Oryza sativa* L. ssp. *japonica*) were used as a source to isolate promoters and genes and for plant transformation. Mature, non-dormant seeds were sterilized and germinated in 1/2 MS medium under a light/dark cycle of 16 h/8 h at 28°C for at least 10 days. Rice seedlings at the trifoliate stage were transferred to plastic buckets with soil at 30°C during the day and 20°C at night in a greenhouse.

### Isolation and Sequence Analysis of the Promoter of *OsUAH*

According to the genomic sequence of *OsUAH* (*LOC_Os12g40550*), the region from 2000 bp upstream to 100 bp downstream of the transcription initiation site for this gene was predicted to be the promoter region (P*_OsUAH_*, as showed in Supplementary Sequence). P*_OsUAH_* was PCR-amplified from rice genomic DNA using gene-specific primers. To identify potential functional elements, the full-length sequence of P*_OsUAH_* was analyzed with the PLACE^1^ and Plant-PAN^2^ software packages as previously described (Luo et al., 2013).

### Promoter-*GUS* Chimeric Vector Construction and Generation of Transgenic Rice Plants

The 5′ deletions of P*_OsUAH_* at positions –1227, –717, –522, –420, and –137 were generated by PCR amplification using different forward primers and a single downstream primer. A *Hin*dIII restriction site was introduced into the forward primers, and an *Eco*RI restriction site was introduced into the reverse primer. The full-length promoter and five deleted derivatives were cut by *Hin*dIII and *Eco*RI and then inserted into the plant transforming binary vector PCAMBIA1391 upstream of the *GUS* coding sequence. The corresponding plasmids were designated as P*_OsUAH_*, P*_Tru1_*, P*_Tru2_*, P*_Tru3_*, P*_Tru4_*, and P*_Tru5_* according to the position at the 5′ end.

Site-specific mutation was performed using the Quick Change Site-Directed Mutagenesis Kit (Transgene, China). The pEASY-T plasmid containing the P*_Tru1_* fragment was used as the PCR template. The obtained mutated construct was cut by *Hin*dIII and *Eco*RI and ligated into PCAMBIA1391. The corresponding plasmid was designated as P*_Tru1_*-M.

To construct potential gain-of-function vectors, the sequence of the CaMV 35S promoter from –46 to +1 (mini 35S) was amplified and inserted into PCAMBIA1391 upstream of the *GUS* coding sequence. The obtained construct was named P*_mini_* and used as a control. A 103-bp fragment that was located in the region from –522 to –420 of P*_OsUAH_* was obtained by PCR using sequence-specific primers with a *Hin*dIII site and an *Eco*RI site. After digestion, the fragment was inserted into P*_mini_* to obtain the recombinant plasmid P*_103bp-mini_*. Full-length P*_OsUAH_* was also fused to P*_mini_* as a positive control (construct P*_OsUAH-mini_*). All primer sequences that were used are listed in Supplementary Tables S1–S10.

The binary constructs were introduced into the *Agrobacterium tumefaciens* strain EHA105. The rice transformation constructs that were used contained the *HPT* gene under the control of the 35S promoter to enable hygromycin-based plant selection. Embryonic calli from the mature rice seeds (*Oryza sativa* L. ssp. *Japonica*) were transformed by co-cultivation, selected with 50 mg/l hygromycin, and used to regenerate transgenic plants as previously described (Duan et al., 2012). The single-copy transgenic lines were screened using the real-time PCR method as described (Yang et al., 2005), and at least four independent T2 lines were selected for further analysis.

### Stress Treatments

To assess the expression levels of the *OsUAH* gene under temperature stress, 10-days-after-germination (DAG) seedlings on agar plates were placed in a growth chamber at constant temperatures of 4 or 42°C under a light/dark cycle of 16 h/8 h. The seedlings were incubated in 1/2 MS solution containing 250 mM NaCl for salt treatment and 100 μM ABA for ABA treatment. For drought stress, the seedlings were dried at 40% relative humidity. Then, the samples were harvested at 0, 4, 8, 12, and 24 h and frozen in liquid nitrogen for RNA extraction.

To analyze the response of P*_OsUAH_* to LT stress at different temperatures, 10-DAG seedlings on agar plates were placed in growth chambers at 4, 10, and 15°C. The control seedlings were grown under the same conditions but at 30°C. The samples were harvested at 0, 4, 8, 12, and 24 h. Mature plants at 60 DAG were treated for 24 h at 4°C, after which the roots, stems and leaves were collected. To analyze the response to LT stress, transgenic plants of truncation and mutation constructs were treated for 24 h at 4°C as above.

### RNA Isolation and qRT-PCR Analysis

The total RNA was extracted from rice using the RNAprep Pure Plant Kit (TIANGEN, China) in accordance with the manufacturer’s instructions. To amplify the corresponding genes, cDNAs were synthesized with random primers using the FastQuant RT Kit (TIANGEN, China) as the template for the qRT-PCR. Real-time quantitative PCR was performed using an ABI PRISM 7500 real-time PCR system (Applied Biosystems, USA) with SYBR Green (TIANGEN, China). The real-time PCR conditions were 95°C for 10 min, followed by 40 cycles of 15 s at 95°C and 1 min at 60°C. The qRT-PCR reactions were performed in triplicate for each cDNA sample. The *ACTIN* gene was used as an internal control, and the relative expression levels were determined in accordance with standard protocols (Livak and Schmittgen, 2001). The expression difference were statistically determined by a on-side paired *t*-test.

### Histochemical GUS Staining

The histochemical localization of GUS activity in transgenic plants was performed as previously described (Wu et al., 2003). The samples were incubated in GUS staining solution (50 mM sodium phosphate at pH 7.0, 10 mM Na_2_-EDTA, 0.1% Triton X-100, 1 mg/ml X-Gluc) at 37°C for 24 h after 15 min of vacuum filtration. After staining, the samples were fixed in 70% ethanol, and photographs were taken under a dissecting microscope.

### Generation of Yeast Reporter Strains for One-Hybrid Screening

For the one-hybrid assay, three tandem copies of the fragment from –443 to –418 of P*_OsUAH_* was synthesized as a bait. In addition, a two-base-substitution fragment and a five-base-substitution fragment at the CRT/DRE element were used as controls. The sequences are shown in Supplementary Table S7. The above three fragments were separately digested with *Hin*dIII and *Sal*I and inserted into the plasmid pAbAi. The obtained recombinant bait plasmids were recognized as pAbAi-E1, pAbAi-E1m2, and pAbAi-E1m5. After digestion with *Bbs*I, the linearized bait plasmids were transformed into yeast strains according to the method that is described in the Matchmaker^TM^ Gold Yeast One-Hybrid Library Screening System Kit (Clontech, USA).

To analyze the CRT/DRE-element-binding activity of OsCBFs, the ORFs of the five rice OsCBF transcription factors *OsCBF1, OsCBF2, OsCBF3, OsCBF4*, and *OsDREB1B* were cloned from the cDNA of *Japonica* rice and were inserted into a GAL4 AD backbone. The pGAD-OsCBF plasmids were then transformed into yeast strains that were integrated with a reporter vector via the LiAc yeast transformation method. The empty AD vector was used as a negative control.

### Purification of Bacterially Expressed Proteins and Electrophoretic Mobility Shift Assay (EMSA)

The pGEX-4T-1 bacterial expression vector system was used to produce a fusion protein with glutathione *S*-transferase (GST). To obtain the fused GST-OsCBF3 protein, the recombinant plasmid was transformed into *Escherichia coli* [Rosetta 2 (DE3) pLysS strain]. The recombinant GST-OsCBF3 protein was induced with 0.4 mM isopropyl-β-D-thiogalactopyranoside for 20 h at 20°C. Protein purification of the GST fusion protein from bacterial extracts was achieved by affinity chromatography with Glutathione Sepharose 4B Resin (GE Healthcare) following the instruction of the manufacturer. Cells carrying the pGEM-4T-1 empty vector were processed as negative controls in an identical manner.

For Electrophoretic Mobility Shift Assay (EMSA), complementary single-stranded oligonucleotides of the E2 probe and its mutants were synthesized, labeled with biotin, and annealed to make probes (Supplementary Table S10). EMSAs were performed using biotin-labeled double-stranded DNA probes with the Light-Shift Chemiluminescent EMSA Kit (Thermo-Fisher Scientific, USA) according to the manufacturer’s instructions.

### *Trans*-activation Experiment with Transgenic Rice

Effector plasmids were constructed with DNA fragments containing *OsCBF1, OsCBF2, OsCBF3, OsCBF4*, and *OsDREB1B* coding regions that were under the control of the maize *UBI* promoter. The effector constructs used *PMI* as a selected marker gene to enable mannose-based plant selection. The transgenic plants were generated, and the expression of the corresponding *CBF* genes was examined using qRT-PCR assays. The overexpressing lines were crossed with three independent single-copy P*_Tru1_* and P*_Tru1_*-M reporter lines containing the *HPT* marker gene as described above. The crossed plants were obtained by hygromycin and mannose double selection.

## Results

### Identification the LT Induction of *OsUAH*

**FIGURE 1:**
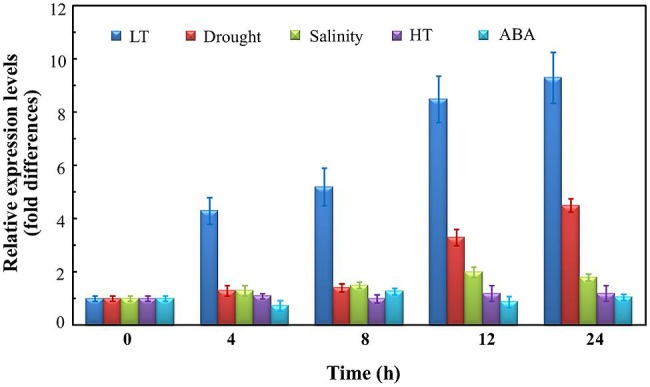
**Quantitative real-time PCR (qRT-PCR) analysis of the *OsUAH* transcripts under various stress conditions.** Rice seedlings at 10 days-after-germination (DAG) were treated with low-temperature (LT; 4°C), high temperature (HT; 42°C), drought (dried at 40% relative humidity), salt (250 mM NaCl) and abscisic acid (ABA; 100 μM ABA). The samples were collected on a time course for RNA isolation and subsequent qRT-PCR analysis. *ACTIN* was used as an internal control. The values are the means ± SD of three independent biological experiments.

To identify the stress response of *OsUAH*, the transcript levels were monitored in time-course treatments of drought, LT, high temperature (HT), salinity, and ABA. As shown in **Figure 1**, *OsUAH* was greatly induced by the cold treatment. The *OsUAH* transcript began to accumulate after 4 h of cold stress treatment and increased in a time-dependent manner. After 24 h of incubation at 4°C, the mRNA level of *OsUAH* was 9.14-fold relative to that of the untreated control. Cool stress (10–15°C) is the most frequently abiotic stress during the early growth stage of rice. Similar with the response to cold stress, *OsUAH* transcripts were also accumulated by the 10 and 15°C incubations (Supplementary Figure S1). In contrast, neither HT, salinity stress nor ABA treatment upregulated the expression of *OsUAH* at any tested time point (**Figure 1**). Under water deficit, *OsUAH* expression did not transcriptionally respond to drought stress in a short period (4 and 8 h air-dry treatment); however, 3.4-fold and 4.2-fold inductions were observed after 12 and 24 h of stress incubation, respectively (**Figure 1**).

To precisely investigate the LT induction of *OsUAH*, the sequence of the predicted promoter (P*_OsUAH_*) with a length of 2100 base pairs (bp) was isolated from the rice (*Oryza sativa* L. cv. Nipponbare) genome and contains 2000 bp immediately upstream and 100 bp downstream (–2000/+100) of the transcription initiation site. The DNA fragment was inserted into a PCAMBIA 1391 vector, generating a rice transformation construct with P*_OsUAH_* driving the *GUS* reporter gene. A total of 28 independent transgenic lines were generated. Among these lines, six single-copy transgenic lines harboring the P*_OsUAH_*::*GUS* construct were screened and selected to determine the expression pattern of P*_OsUAH_*.

**FIGURE 2:**
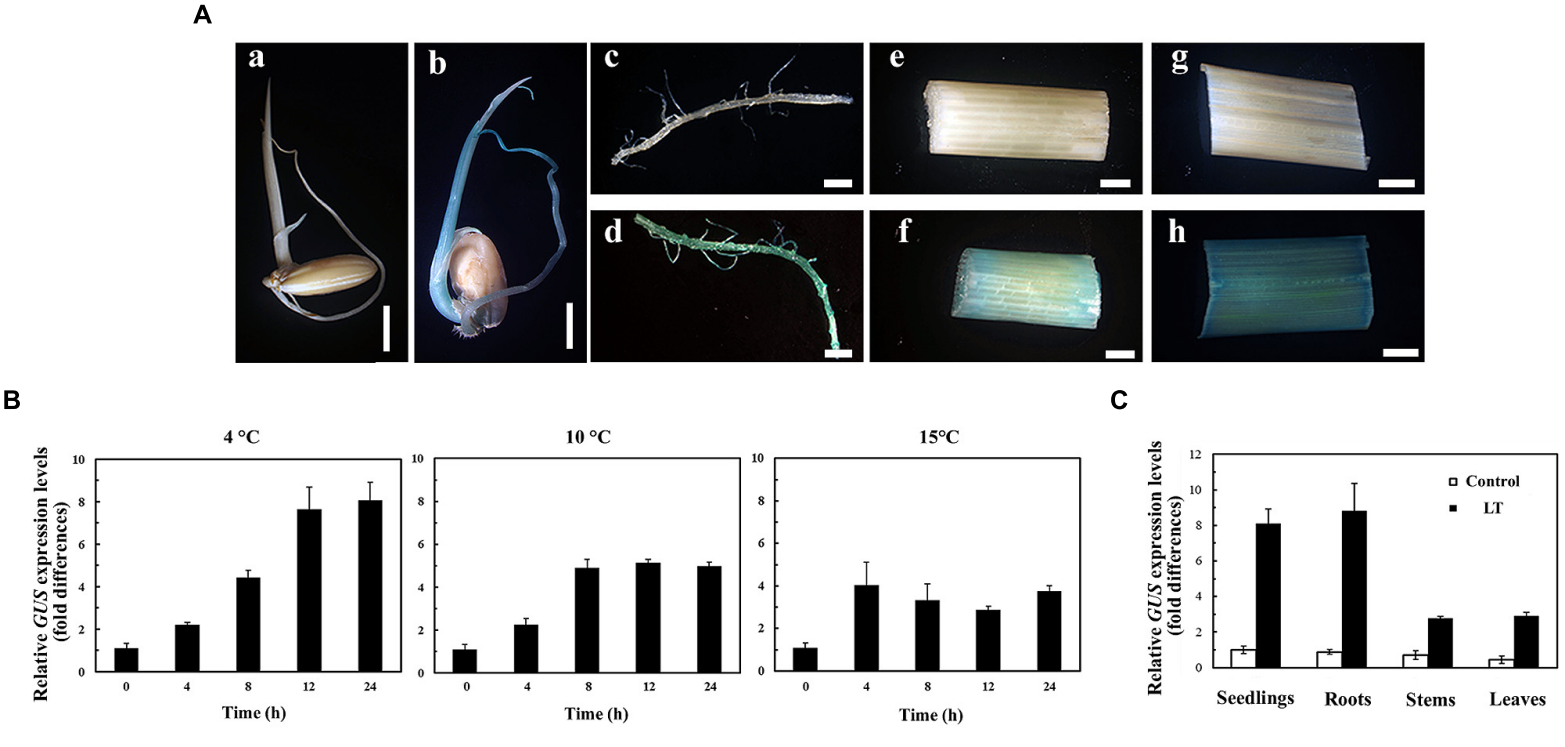
**The LT response of P*_OsUAH_*. (A)** The GUS staining of the transgenic rice harboring the P*_OsUAH_*::*GUS* construct. 5-DAG seedlings **(a,b)**; and roots **(c,d)**, stems **(e,f)**, and leaves **(g,h)** of 60-DAG plants were incubated under normal growth conditions **(a,c,e,g)** or LT (4°C) treatment for 24 h **(b,d,f,h)**. **(B)** Analysis of the promoter activity induction under different LT conditions. Seedlings at 10 DAG were treated with 4, 10, and 15°C for 24 h. *GUS* transcripts were measured by qRT-PCR. **(C)** qRT-PCR analysis of *GUS* induction in different organs of transgenic rice. The 60-DAG plants were treated with LT (4°C) for 24 h, after which the mature leaves, stems and roots were collected. *GUS* induction was examined with a parallel experiment on 10-DAG seedlings. The *GUS* mRNAs of the untreated transgenic plants were set to one. The values are the means ± SD of six independent biological experiments.

The activity of P*_OsUAH_* was first examined by GUS histochemical staining. Under normal growth conditions, the GUS staining could not be detected in any of transgenic plants, regardless of tissues or developmental stages (**Figure 2A**). However, after LT treatment, obvious blue staining was observed not only in 5-DAG seedlings, but also in tissues of plants at the booting stage (60 DAG). The LT response of P*_OsUAH_* was also determined by quantitative reverse real-time PCR (qRT-PCR). The *GUS* transcript was significantly induced after 4 h of incubation at 4°C (*p* < 0.05). The expression gradually increased in a time-dependent manner, reaching 8.08-fold at 24 h. The LT responses of the promoter were also examined by the incubation at 10 and 15°C. As shown in **Figure 2B**, the activities of the promoter could be significantly induced after 4 h of incubations (*p* < 0.05), after which the activities slowly increased or remained relatively constant (**Figure 2B**). To further investigate the expression pattern of P*_OsUAH_, GUS* levels were individually measured in different tissues of 60-DAG plants. The transcripts were markedly increased by LT stress (4°C incubation for 24 h), while the fold-induction level in the roots (11.01-fold) was relatively higher than that in the leaves (5.76-fold) and stems (3.94-fold), (**Figure 2C**).

### Identification of the Minimal Promoter Region for LT-Inducible Expression

**FIGURE 3:**
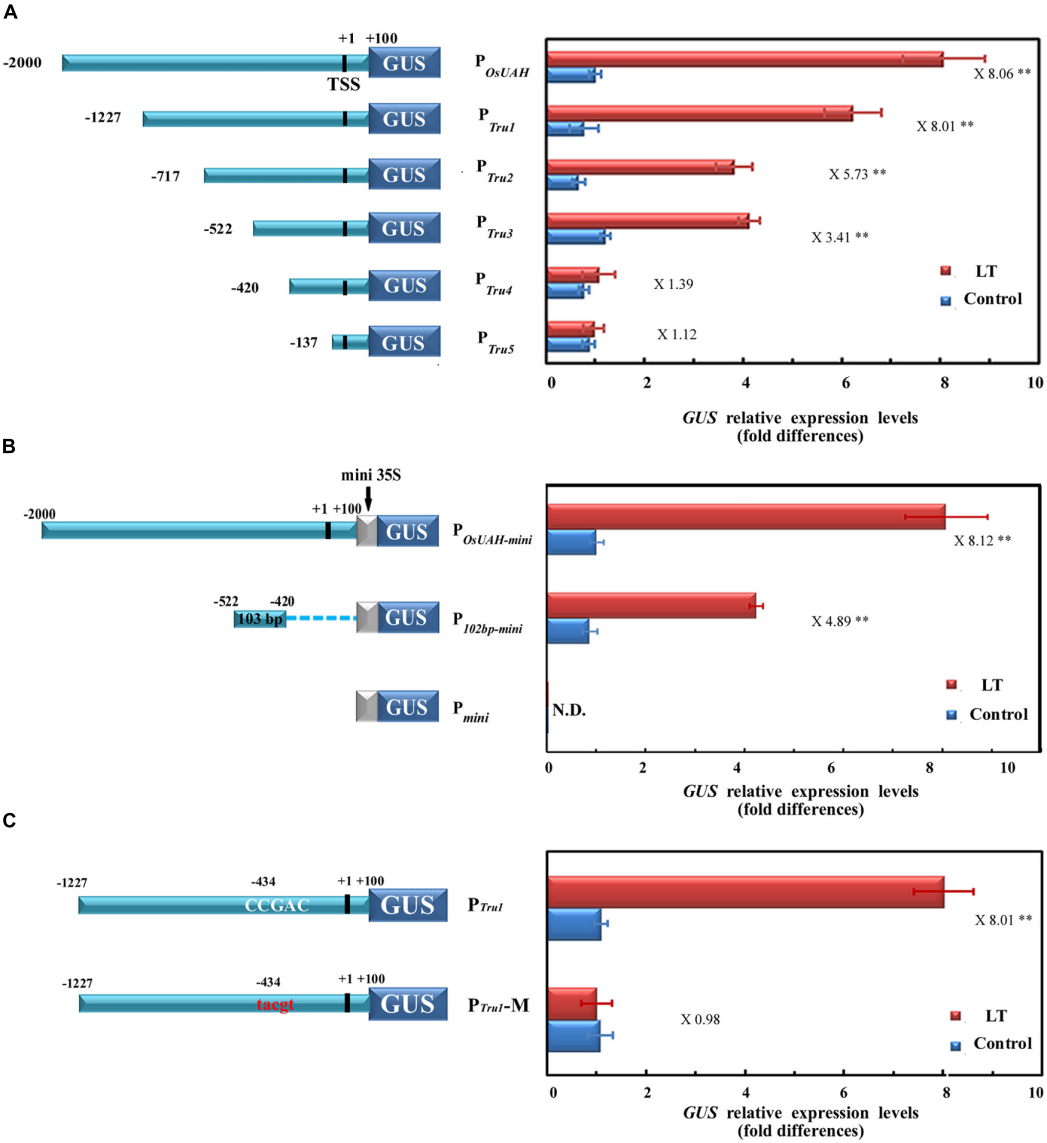
**Activity analysis of P*_OsUAH_* for determining the minimal region of LT induction. (A)** Deletion analysis of P*_OsUAH_*. Left, schematic diagrams of serial 5′ deletion constructs of P*_OsUAH_*. The locations of the 5′ ends of the five fragments of P*_OsUAH_* are indicated. LT stress was applied by incubating 10-DAG seedling at 4°C for 24 h. The activities were then compared by analyzing *GUS* transcript abundance in corresponding transgenic populations. The *GUS* mRNAs of untreated full-length P*_OsUAH_* transgenic plants were averaged and set to one. **(B)** Identification of the 103-bp region that was responsible for the LT response. The 103-bp sequence between position –522 and –420 was linked at the 5′ region upstream of the mini35S promoter region as the P*_103bp-mini_* construct. Full-length P*_OsUAH_* was also fused to the mini35S to generate P*_OsUAH-mini_* as a positive control, and the empty P*_mini_* construct was used as a negative control. After generating transgenic plants, their LT induction abilities were examined by determining *GUS* transcriptional expression. The *GUS* mRNAs of untreated P*_OsUAH-mini_* transgenic plants were averaged and set to one. **(C)** Identification of the *cis*-element that is responsible for the LT response. Left, Schematic diagrams of the P*_Tru1_* construct and the mutated P*_Tru1_* (P*_Tru1_*-M) containing a mutation on the core C-repeat/dehydration-responsive (CRT/DRE) element (from the conserved CCGAC to the irrelevant 5-bp sequence TGCGA). Right, the corresponding promoter activity as determined in the stably transformed transgenic rice plants. The *GUS* mRNAs of untreated P*_Tru1_* transgenic plants were averaged and set to one. The ratio of the promoter activity with LT stress to that without LT stress (induction ratio) is shown at the far right. TSS: transcriptional start site. The relative expression levels were averaged from at least four independent biological experiments. The significant difference between the LT-stress and non-stress conditions was analyzed using a one-side paired *t*-test (^∗∗^*p* < 0.01).

In an attempt to define the specific regions of P*_OsUAH_* that are involved in LT-inducible expression, a series of 5′ deletions in the 2100 bp promoter were generated (**Figure 3A**). After fusion to a *GUS* reporter gene, five truncation fragments were separately transformed into rice. For each construct, at least four independent single-copy transgenic lines were selected, and the *GUS* mRNA levels were quantitatively assayed. All of the truncation fragments had similar expression levels as those of the full-length P*_OsUAH_* under normal growth conditions. Under a LT treatment at 4°C for 24 h, the *GUS* levels were significantly induced in the constructs containing deletions of the distal part of the full-length sequence to positions –1227 (construct P*_Tru1_*), –717 (construct P*_Tru2_*), and –522 (construct P*_Tru3_*) from the transcription initiation site, while P*_Tru1_* had a statistically similar induction fold as that of P*_OsUAH_*, and the induction levels of P*_Tru2_* and P*_Tru3_* decreased (**Figure 3A**). In contrast, the LT inductions were completely lost in the constructs containing deletions to positions –420 (construct P*_Tru4_*) and –137 (construct P*_Tru5_*), (**Figure 3A**), suggesting that the 103-bp fragment between positions –522 and –420 is essential for the LT induction of P*_OsUAH_*.

To confirm that the region between positions –522 and –420 plays a crucial role in P*_OsUAH_*, the 103-bp fragment was separated and fused to a mini35S promoter (construct P*_103bp-mini_*) and then linked to the binary vector to drive *GUS*. Meanwhile, full-length P*_OsUAH_* was also fused to the mini35S promoter as a positive control (construct P*_OsUAH-mini_*). The constructs, as well as the empty mini35S::*GUS* vector (P*_mini_*), were stably transformed into rice. As shown in **Figure 3B**, both P*_OsUAH-mini_* and the P*_103bp-mini_* were still able to be induced by LT treatment, although the fold-induction level of P*_OsUAH-mini_* (8.1-fold) was relatively higher than the level of P*_103bp-mini_* (4.89-fold). In addition, the expression of P*_mini_* was not altered by LT stress. These results indicate that the 103-bp minimal region is sufficient to activate the LT-induced expression and should contain *cis*-elements, which are responsible for the LT stress response.

To further identify the element(s) that are responsible for LT induction, a sequence analysis was performed on the 103-bp fragment between positions –522 and –420. A CCGAC element, which is the core sequence of the C-repeat/dehydration-responsive (CRT/DRE) element, was located at position –434, closely associated with the expression of LT or drought stresses. To determine whether the CRT/DRE element is involved in LT induction, the CCGAC sequence was substituted *in situ* in the construct P*_Tru1_* with an irrelevant 5-bp sequence, TGCGA, generating the construct P*_Tru1_*-M. Under normal conditions, the activity of P*_Tru1_*-M remained the same as that of the non-mutated P*_Tru1_* in the corresponding transgenic plants. However, the *GUS* mRNA accumulations by LT treatment were fully abolished in all of the tested P*_Tru1_*-M transgenic rice compared to the 8.01-fold induction in plants harboring P*_Tru1_* (**Figure 3C**). These results suggest that this element is essential to the LT induction of *OsUAH*.

### OsCBF3 Specifically Binds to the CRT/DRE Element in the Minimal Region of P*_OsUAH_*

**FIGURE 4:**
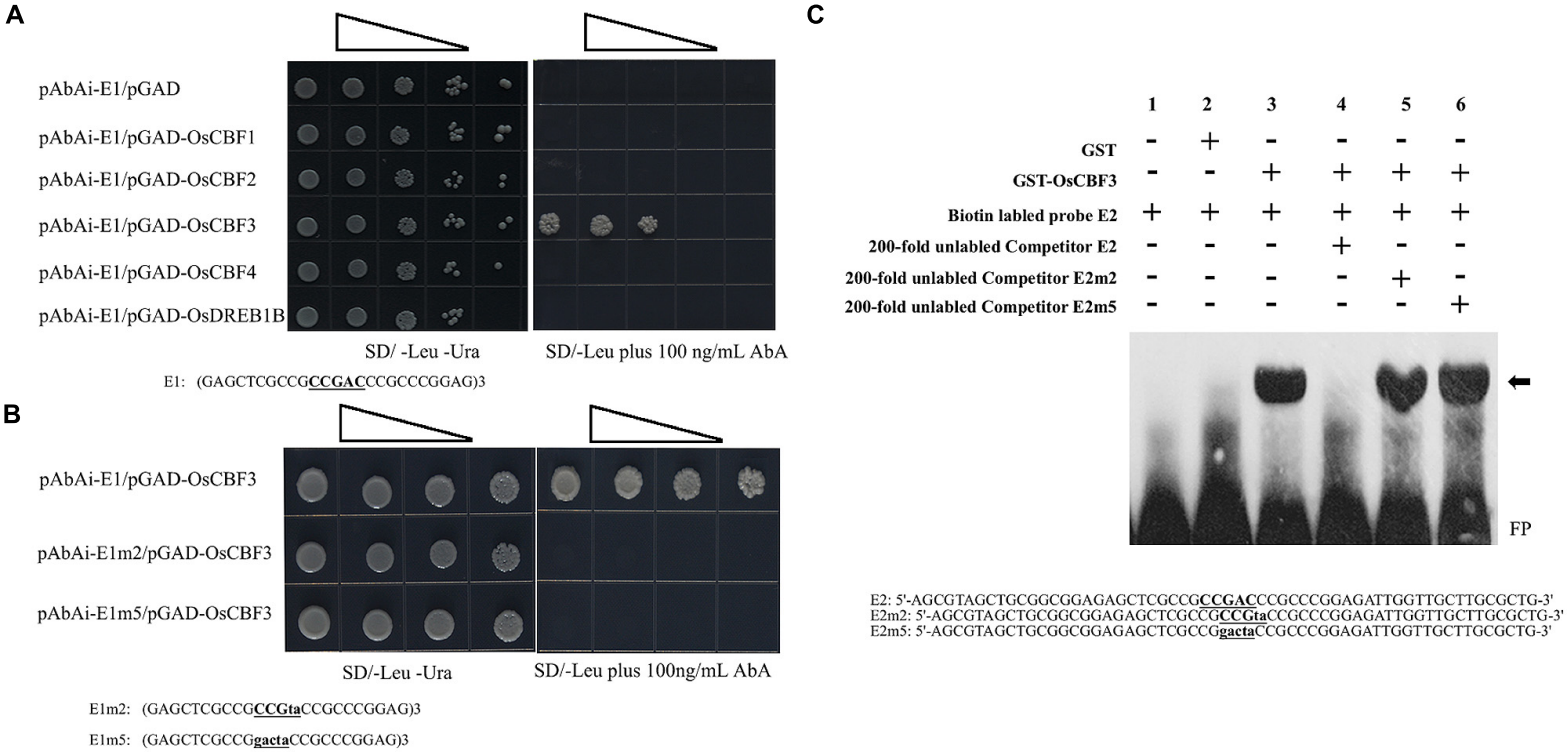
**The core CRT/DRE element specifically interacted with OsCBF3. (A)** Yeast one-hybrid analysis of the binding of rice CBFs to P*_OsUAH_*. Upper, OsCBF3 specifically interacted with P*_OsUAH_*. Yeast cells were co-transformed with a bait vector containing three consecutive repeats of the 25-bp P*_OsUAH_* fragment surrounding the core CRT/DRE element (underlined) and fused to an *AUR1-C* reporter gene (pAbAi-E1) and with a prey vector containing a CBF transcription factor coding sequence that was fused to a *GAL4* activation domain (pGAD-CBF). The binding of the *GAL4* activation domain (pGAD empty vector) to the P*_OsUAH_* fragment (pAbAi-E1) was used as a negative control. Lower, OsCBF3 interacted with the CRT/DRE element in P*_OsUAH_*. The bait vector carrying the corresponding fragment and the prey pGAD-OsCBF3 vector were co-transfected into yeast cells. The fragments: E1, Three copies of the P*_OsUAH_* fragment; E2m, two-base mutation of the CRT/DRE element in E1; and E5m, five-base mutation of the CRT/DRE element in E1. The cells were grown in liquid media to an OD_600_ of 0.1 (10^-1^) and diluted in a 10× dilution series (10^-2^ to 10^-4^/10^-3^). Of each dilution, 6 μL was spotted onto media that selected for both of the plasmids (SD–Ura–Leu) and selecting for interaction (SD–Leu) and that was supplemented with 100 ng/ml Aureobasidin A (AbA) to suppress the background growth and test the strength of the interaction. **(B)** The Electrophoretic Mobility Shift Assay (EMSA) assays indicate that the glutathione *S*-transferase (GST)-OsCBF3 fusion protein but not GST can specifically bind to the CRT/DRE element (underlined) in the 59-bp P*_OsUAH_* sequence. The probes were incubated with recombinant proteins in 20-μL reactions. **(C)** Lane 1, 20 fmol of the labeled wild-type probe (E2) alone. Lane 2, 20 fmol of labeled E2 probe with 1 μg of the recombinant GST protein. Lane 3, 20 fmol of the labeled E2 probe with 1 μg of the recombinant GST-OsCBF3 protein. Lane 4, 20 fmol of the labeled E2 probe with 1 μg of the recombinant GST-OsCBF3 protein and 2 pmol of the unlabeled competitor E2 probe. Lane 5, 20 fmol of the labeled E2 probe with 1 μg of the recombinant GST-OsCBF3 protein and 2 pmol of the unlabeled competitor probe with the two-base mutation on the CRT/DRE element (E2m2). Lane 6, 20 fmol of the labeled E2 probe with 1 μg of the recombinant GST-OsCBF3 protein and 2 pmol of the unlabeled competitor probe with the five-base mutation on the CRT/DRE element (E2m5). The mutation sites are labeled with lower case letters. The arrow indicates the up-shifted bands. FP, Free probe. GST is from the expression vector pGEM-4T-1.

The interaction between the CRT/DRE elements with the CBF transcript factors has been identified in various plants (Sakuma et al., 2002; Qin et al., 2004). In the rice genome, 10 putative *CBF/DREB* homologs (*OsDREB1A* – *OsDREB1J*) have been identified. Five of these homologs, *OsDREB1C*/*OsCBF1* (*LOC_Os06g03670*), *OsDREB1F*/*OsCBF2* (*LOC_Os01g73770*), *OsDREB1A*/*OsCBF3* (*LOC_Os09g35030*), *OsDREB1D*/*OsCBF4* (*LOC_Os06g06970*), and *OsDREB1B* (*LOC_Os09g35010*), are induced by LT stress (Dubouzet et al., 2003; Liu et al., 2007; Wang et al., 2008). To determine the specific rice CBF factor that could directly target the core CRT/DRE element in the minimal region of P*_OsUAH_*, a yeast one-hybrid assay was used. Three tandem copies of the 25-bp sequence surrounding the CRT/DRE element were synthesized as E1 bait. E1 was fused in front of the reporter gene *AUR1-C*, an antibiotic resistance gene that confers Aureobasidin A (AbA) resistance in yeast. Meanwhile, five *OsCBF/DREB1* genes were separately cloned and fused to a GAL4 activation domain (AD) as preys. After being co-transformed with the promoter and individual *CBF*, only the yeast cells harboring *OsCBF3* and E1 could grow on the 100 ng/ml SD/-Leu/AbA medium, while the co-transformant with other rice CBFs could not survive under AbA selection. These observations indicate that OsCBF3 is the only candidate binding to P*_OsUAH_* among the tested rice CBF members in yeast (**Figure 4A**).

To explore whether the element is the binding site of OsCBF3 in the promoter, two site-directed mutations were performed on the core sequence of E1, generating a two-base substitution (E1m2: CCGta) and a five-base substitution (E1m5: gacta). The AD-OsCBF3 yeast cells harboring baits with either E1m2 or E1m5 could not grow on the leucine dropout medium that was supplemented with 100 ng/ml AbA. In contrast, the growth of yeast cells with OsCBF3 and wild-type E1 was not inhibited by AbA (**Figure 4B**). These results suggest that the core CRT/DRE element is the specific binding site of OsCBF3 in the P*_OsUAH_* fragment.

To further confirm that OsCBF3 binds to P*_OsUAH_* at the CRT/DRE element, purified full-length OsCBF3 protein was obtained using a GST- fusion purification system and used to perform Electrophoretic Mobility Shift Assay (EMSA). As shown in **Figure 4C**, the GST-OsCBF3 protein bound to a 59-bp E2 probe from the minimal region of P*_OsUAH_* that contained the CRT/DRE element. A competition EMSA was performed in parallel with wild-type and mutant unlabeled E2 probes. **Figure 4C** showed that excessive wild-type E2 probe could compete with the labeled probe, but the same amount of unlabeled mutant E2 probe with the two-base substitution (E2m2) or five-base substitution (E2m5) on the core sequence of E2 did not, suggesting that OsCBF3 protein could bind specifically to P*_OsUAH_* at the CRT/DRE element *in vitro*.

### OsCBF3 *Trans-*activates the Expression of P*_OsUAH_* in a CRT/DRE Element-Dependent Manner

**FIGURE 5:**
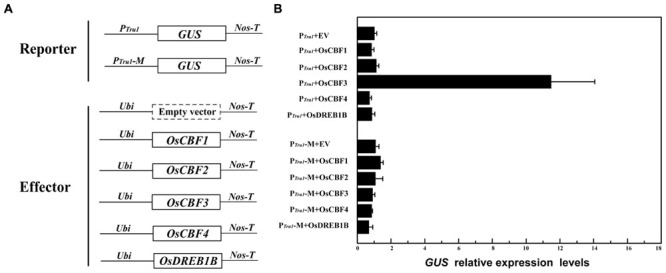
**OsCBF3 specifically *trans*-activated P*_OsUAH_ in planta*. (A)** Schematic diagrams of the vectors. The *CBFs* were driven by a maize ubiquitin promoter as effectors, and the truncation promoter P*_Tru1_* and the CRT/DRE element mutated derivate P*_Tru1_*-M (described in **Figure 3**) were fused to drive *GUS* as reporters. **(B)** qRT-PCR analysis of *GUS* expression. The *GUS* mRNAs of the crossed transgenic plants containing P*_Tru1_* plus the empty effector vector were set to one. The relative expression levels were averaged from three independent biological experiments.

To detect the *in vivo* interactions between CBFs and P*_OsUAH_*, promoter *trans*-activation assays were performed. The constructs are schematically represented in **Figure 5A**. Because the truncation fragment at position –1227 (P*_Tru1_*) has a similar expression pattern as that of the full-length promoter P*_OsUAH_*, P*_Tru1_* and the above-described P*_Tru1_*-M containing the mutated CRT/DRE element were used as reporters. Five LT-responsive rice *CBF* members were overexpressed by a maize ubiquitin promoter as effectors. The transgenic plants containing the effector were regenerated and crossed with the single-copy reporter plant lines. The crossed plants harboring P*_Tru1_* and *OsCBF3* resulted in an 11.47-fold increase in *GUS* mRNA accumulation compared to the background level of the cross of P*_Tru1_* and an empty-effector vector, whereas the co-expression of other *CBF* members exhibited the same *GUS* levels as that of the empty-effector vector (**Figure 5B**). These results indicate that P*_Tru1_* only could be activated by *OsCBF3*, which is consistent with the results of the previous binding activity assay in yeast. None of the effectors could induce the expression of the mutated P*_Tru1_*-M reporter, suggesting that the *trans*-activation of P*_OsUAH_* by *OsCBF3* depends on the CRT/DRE element.

## Discussion

Nitrogen supplementation plays a critical role in the utilization of absorbed light energy and photosynthetic carbon metabolism (Kato et al., 2003; Huang et al., 2004). Adequate nitrogen supplementation mitigates the damages of abiotic stresses (Huang et al., 2004; Waraich et al., 2011), while a high risk of photo-oxidative damage is expected in the nitrogen-deficient leaves under stress (Verhoeven et al., 1997). Purine catabolism is important for nitrogen recycling. In this study, we identified *OsUAH*, the catalyzer of the final step of the ureide-degrading reactions of purine ring catabolism (Werner et al., 2010), as an LT-responsive gene. This result has been confirmed by independent transcriptome analyses (Maruyama et al., 2011; Zhang et al., 2012; Shaik and Ramakrishna, 2013). In addition to previous evidences of the LT induction of other catabolic genes, our results suggest that ureide degradation might be critical for nitrogen redistribution in the LT adaption of plants. Furthermore, our results indicate the *OsUAH* has a relatively higher induction level in the roots than in photosynthetic tissues, suggesting a potential nitrogen redistribution pattern in response to stress. Various studies have emphasized that nitrogen metabolism genes are regulated by LT stress (Cui et al., 2005; Pageau et al., 2006; Zhu et al., 2007; Robinson and Parkin, 2008; Maruyama et al., 2009; Zhang et al., 2012), while the underlying mechanisms have seldom been reported. The hydrolase genes of purine catabolism, e.g., *XDH* and *ALN*, are regulated by ABA (Hesberg et al., 2004; Alamillo et al., 2010). However, we found that the expression of *OsUAH* was not induced by exogenous ABA (**Figure 1**), and our data indicated the LT-induction of *OsUAH* was associated with a CRT/DRE element of P*_OsUAH_*. The evidence from yeast assays and the *in vitro* interaction analysis demonstrate that this element physically binds to the CBF transcriptional factor OsCBF3. Further assays demonstrated that P*_OsUAH_* activity could be upregulated by overexpressing *OsCBF3 in vivo*. These data suggest that the regulation of *OsUAH* is involved in a CBF-related LT-response pathway. In addition to LT stress, *OsUAH* is also induced by drought stress, which is consistent with previous transcriptional profile studies (Degenkolbe et al., 2009; Gao et al., 2009). Because OsCBF3 is a transcription activator of cold- and drought-responsive gene expression, the induction of *OsUAH* could be explained by the interaction between the CRT/DRE element of P*_OsUAH_* and OsCBF3. In most plants, the CBF pathway of the drought and LT stress responses is ABA-independent (Shinozaki et al., 2003; Yamaguchi-Shinozaki and Shinozaki, 2006). Therefore, our results suggest the *OsUAH* might respond to environmental stress in a distinct way compared to other ureide metabolism genes.

C-repeat-binding factor transcriptional factors are involved in the activation of LT-responsive genes by interacting with CRT/DRE elements in the promoter. Most LT-responsive genes have multiple CRT/DRE elements in their promoters. However, the element that is located at –434 bp is an unique copy in the P*_OsUAH_* sequence. Our assays identified a synthetic promoter with a 103-bp fragment containing an element that exhibited similar LT-induction activity as that of P*_OsUAH_*, whereas the deletion or mutation of this element thoroughly abolished this induction, indicating that this element is sufficient to determine the LT response of P*_OsUAH_*. In addition, the deletion between –1227 and –717 of P*_OsUAH_* significantly decreased LT induction level. However, the bioinformatic screening did not detect any potential stress response element in this region. Therefore, it is likely that an enhancer exists in the area that facilitates the intensity of the promoter.

C-repeat-binding factor/DREB1 is a small subfamily of the APETALA2/Ethylene response factor (AP2/EREBP) family of transcription factors. In *Arabidopsis*, this subfamily contains six members (Nakano et al., 2006; Lata and Prasad, 2011). Extensive studies have demonstrated that three tandem-distributing *CBF* genes, *CBF1, CBF2*, and *CBF3* (also known as *DREB1B, DREB1C*, and *DREB1A*, respectively), play a central role in the transcriptional regulation of LT responsive genes. Five rice *CBFs* were identified as induced by cold stress, and overexpressing any of them can result in the enhancement of plant cold tolerance (Dubouzet et al., 2003; Ito et al., 2006; Liu et al., 2007; Qin et al., 2007; Wang et al., 2008). CBFs have highly conserved functional domains. In most cases, the cold-induced CBFs have redundant activities in plant development and stress adaption as identified in gain-of-function experiments by activating the same gene clusters (Gilmour et al., 2004; Zhou et al., 2010; Lata and Prasad, 2011). However, little evidence had suggested that each individual CBF might regulate different targets and thus separately contribute to transcriptome alteration in response to cold. In *Arabidopsis, CBF1* and *CBF3* RNAi transgenic plants impair the induction of cold-responsive genes; in contrast, the cold tolerance of the loss-of-function mutant of *CBF2, cbf2*, is enhanced by upregulating *CBF1* and *CBF3* (Novillo et al., 2004), indicating that the functional differentiation despite the gain-of-function modification leads to exactly same phenotype (Gilmour et al., 2004). In this study, we first demonstrated that P*_OsUAH_* exclusively bound to OsCBF3 in yeast. Then, we found that OsCBF3 could activate P*_OsUAH_* by interacting with the CRT/DRE element. Meanwhile, the other four rice *CBFs* did not affect the promoter activity, although their expression was successfully enhanced similar to *OsCBF3* (Supplementary Figure S2). These results suggest that rice CBFs also regulate different objectives, similar to *Arabidopsis* homologs, and that their function does not fully overlap. Although the overexpression of rice *CBFs* exhibited a similar stress adaption phenotype (Dubouzet et al., 2003; Ito et al., 2006; Wang et al., 2008), it was reasonable to expect a diversity of function when individual CBF mutant of rice was generated. Furthermore, the rice CBF family has a more complicated structure than that of *Arabidopsis* or other identified dicot plants. In this study, the activity of P*_OsUAH_* was not detected before or after LT induction in a transient expression system of tobacco leaves. However, P*_OsUAH_* could be activated via the co-agroinjection of OsCBF3 in tobacco (Supplementary Figure S3). These results indicate that tobacco may lack functional homologs of OsCBF3, suggesting that the OsCBF3-specific *trans*-activated regulation might be unique between species.

## Conflict of Interest Statement

The authors declare that the research was conducted in the absence of any commercial or financial relationships that could be construed as a potential conflict of interest.

## Footnotes

1 http://www.dna.affrc.go.jp/PLACE/

2 http://plantpan.mbc.nctu.edu.tw/index.php
