# Supplementary material for: Low-Temperature-Induced Expression of Rice Ureidoglycolate Amidohydrolase is Mediated by a C-Repeat/Dehydration-Responsive Element that Specifically Interacts with Rice C-Repeat-Binding Factor 3
Source: Front Plant Sci. 2015 Nov 13;6:1011. doi: 10.3389/fpls.2015.01011 (PMC4643140; doi:10.3389/fpls.2015.01011)
Supplement: Supplementary file 1 [file Table_1.DOC]

Supplementary Material

**Low-temperature-induced expression of rice *ureidoglycolate amidohydrolase* is mediated by a C-repeat/dehydration-responsive element that specifically interacts with rice C-repeat-binding factor 3**

Juan Li, Rui-Ying Qin, Hao Li, Rong-Fang Xu, Ya-Chun Yang, Da-Hu Ni, Hui Ma, Li Li, Peng-Cheng Wei^*^, Jian-Bo Yang^*^

**Correspondence:** Peng-Cheng Wei, Email: [weipengcheng@gmail.com](mailto:weipengcheng@gmail.com); or Jian-Bo Yang, Email: yjianbo@263.net.

# Supplementary Figures and Tables

## Supplementary Figures

**Figure S1, qRT-PCR analysis of the *OsUAH* transcripts under cool stress.** 10-DAG seedlings on agar plates were incubated in growth chambers at 10 °C and 15 °C for 4 h and 24 h. The transcripts of *OsUAH* were examined by qRT-PCR assays. *ACTIN* was used as an internal control. The values are the means ± SD of three independent biological experiments.

**Figure S2, qRT-PCR analysis on the rice *CBF* expression level in corresponding effector transgenic lines.** The determined lines were used to cross with the reporter plant. Data bars represent the means ±SD of triplicate measurements. EV: empty vector.

**Figure S3,** **Transient trans-activation assay on P*_Tru1_*in tobacco.** Effector and reporter vector were co-transfected into tobacco leaves by agroinfiltration. The promoter activities were compared by quantitatively determination of GUS activity. Data bars represent the means ±SD of triplicate measurements. N.D.: non detected.

## Supplementary Tables

**Table S1.**  Sequence of oligonucleotides used to create P*_OsUAH_*, truncation constructs

| Primer name | (5’ to 3’, sequences underlined to show endonuclease recognition site) | Restriction site | Experiment |
| --- | --- | --- | --- |
| P*_OsUAH_*-FP | AAGCTTTGACTTAGTAATGATAACAACA | *Hin*dIII | For P*_OsUAH_* and P*_OsUAH-mini_* construction |
| F1 | AAGCTTAACCTGGCCATTTCTTCTCATC | *Hin*dIII | For P*_Tru1_* construction |
| F2 | AAGCTTATCTAACTGTGGACTACACGAA | *Hin*dIII | For P*_Tru2_* construction |
| F3 | AAGCTTATCCAAGCTACACCTCCCGCGG | *Hin*dIII | For P*_Tru3_* construction |
| F4 | AAGCTTCTCTCCGCCGCAGCTACGCTAG | *Hin*dIII | For P*_Tru4_* construction |
| F5 | AAGCTTCGCCCACTCCTGGGCCCAGCCC | *Hin*dIII | For P*_Tru5_* construction |
| P*_OsUAH_*-RP | GAATTCGCCCGCGAGGGCAGCGAGGAAG | *Eco*RI | For P*_OsUAH_* and truncation constructs |

**Table S2.**  Sequence of oligonucleotides used to create P*_Tru1_*-M construct

| Primer name | Sequence （5’ to 3’, underlined sequences showed the mutant site） |
| --- | --- |
| P*_Tru1_*-MF | CCAATCTCCGGGCGGACGTACGGCGAGCTC |
| P*_Tru1_*-MR | TACGTCCGCCCGGAGATTGGTTGCTTGCGC |

**Table S3.** Sequence of oligonucleotides used to create gain-of-function constructs

| Primer name | (5’ to 3’, endonuclease recognition sites were underlined) | Restriction site | Experiment |
| --- | --- | --- | --- |
| P*_OsUAH_*-FP | AAGCTTTGACTTAGTAATGATAACAACA | *Hin*dIII | For P*_OsUAH-mini_* construction |
| P*_OsUAH_*-RP | GAATTCGCCCGCGAGGGCAGCGAGGAAG | *Eco*RI |
| P3 | AAGCTTATCCAAGCTACACCTCCCGCGG | *Hin*dIII | For P*_103bp-mini_* construction |
| P4-RP | GAATTCCTAGCGTAGCTGCGGCGGAGAG | *Eco*RI |

**Table S4.** Primers and TaqMan probe sequences used for qRT-PCR in copy number analysis

| Primer name | Primer sequence (5’ to 3’) |
| --- | --- |
| SPS-FP | TCTCCTCGTCCAGTGCTTCTC |
| SPS-RP | TTGGTGGACGCGCTTCTAG |
| SPS-Probe | TET-TCCTCGCAACCGAAC-TAM |
| HPT-FP | CTATTTCTTTGCCCTCGGACGA |
| HPT-RP | GGACCGATGGCTGTGTAGAAG |
| HPT-Probe | FAM-CGCCGATAGTGGAAACCGACGCCC-TAM |

**Table S5.** Primers used to amplify the selected genes for qRT-PCR

| Primer name | Primer sequence (5’ to 3’) |
| --- | --- |
| Actin-FP | CCTGACGGAGCGTGGTTAC |
| Actin-RP | CCAGGGCGATGTAGGAAAGC |
| OsUAH-FP | GCGCTGGGAGGGTTCTGAGGCA |
| OsUAH-RP | CCTCAAGAGCACCCAGGACACC |
| Gus- FP | TACGGCAAAGTGTGGGTCAATAATCA |
| Gus- RP | CAGGTGTTCGGCGTGGTGTAGAG |

**Table S6**. Sequence of oligonucleotides used to construct effectors for *trans*-activation assay

| Primer name | Primer sequence (5’ to 3’) | Restriction site |
| --- | --- | --- |
| OsCBF1 FP1 | AAGCTTatggagtactacgagcaggagg | *Hin*dIII |
| OsCBF1 RP1 | GGTACCGTAGCTCCAGAGTGTGACGTCG | *Kpn*I |
| OsCBF2 FP1 | AAGCTTATGGACACCGAGGACACGTCGT | *Hin*dIII |
| OsCBF2 RP1 | GGTACCGTCCATCCATAGCTTGTAGTCC | *Kpn*I |
| OsCBF3 FP1 | AAGCTTATGTGCGGGATCAAGCAGGAGA | *Hin*dIII |
| OsCBF3 RP1 | GGTACCGTAGCTCCAGAGTGGGACGTCG | *Kpn*I |
| OsCBF4 FP1 | ACTAGTATGGAGAAGAACACCGCCGCCA | *Spe*I |
| OsCBF4 RP1 | GGTACCCCTCCTCCATTGAAAAAGGTGG | *Kpn*I |
| OsDREB1B FP1 | AAGCTTatggaggtggaggaggcggcgt | *Hin*dIII |
| OsDREB1B RP1 | GGTACCGTAGCTCCAGAGCGGCATGTCG | *Kpn*I |

**Table S7.** Sequence of oligonucleotides used for yeast one-hybrid screening

| name | Sequence （5’ to 3’, sequences underlined to show *Hin*dIII/*Sal*I endonuclease recognition site, the DRE/CRT element was labeled by red and mutations were showed by lower case letter） |
| --- | --- |
| E1 | TCGACGAGCTCGCCGCCGACCCGCCCGGAGA |
| E12m | TCGACGAGCTCGCCGCCGtaCCGCCCGGAGA |
| E15m | TCGACGAGCTCGCCGgactaCCGCCCGGAGA |

**Table S8.**  Sequence of oligonucleotides used to create AD-OsCBF constructs

| Primer name | Primer sequence (5’ to 3’) | Restriction site |
| --- | --- | --- |
| OsCBF1-FP2 | CATATGatggagtactacgagcaggagg | *Nde*I |
| OsCBF1 RP2 | GAATTCGTAGCTCCAGAGTGTGACGTCG | *Eco*RI |
| OsCBF2 FP2 | CATATGATGGACACCGAGGACACGTCGT | *Nde*I |
| OsCBF2 RP2 | GAATTCGTCCATCCATAGCTTGTAGTCC | *Eco*RI |
| OsCBF3 FP2 | CATATGATGTGCGGGATCAAGCAGGAGA | *Nde*I |
| OsCBF3 RP2 | GAATTCGTAGCTCCAGAGTGGGACGTCG | *Eco*RI |
| OsCBF4 FP2 | CATATGATGGAGAAGAACACCGCCGCCA | *Nde*I |
| OsCBF4 RP2 | CTCGAGCCCTCCTCCATTGAAAAAGGTG | *Xho*I |
| OsDREB1B FP2 | CATATGatggaggtggaggaggcggcgt | *Nde*I |
| OsDREB1B RP2 | GAATTCGTAGCTCCAGAGCGGCATGTCG | *Eco*RI |

**Table S9.**  Sequence of oligonucleotides used to create GST-OsCBF3

| Primer name | Primer sequence(5’ to 3’) | Restriction site |
| --- | --- | --- |
| OsCBF 3 FP3 | GGATCCATGTGCGGGATCAAGCAGGAGA | *Bam*HI |
| OsCBF 3 RP3 | GAATTCGTAGCTCCAGAGTGGGACGTCG | *Eco*RI |

**Table S10.** Sequence of oligonucleotides used for EMSA assays

| Name | Sequence （5’ to 3’, the DRE/CRT element was underlined and mutations were showed by lower case letter） |
| --- | --- |
| E2 | AGCGTAGCTGCGGCGGAGAGCTCGCCGCCGACCCGCCCGGAGATTGGTTGCTTGCGCTG |
| E2m2 | AGCGTAGCTGCGGCGGAGAGCTCGCCGCCGtaCCGCCCGGAGATTGGTTGCTTGCGCTG |
| E2m5 | AGCGTAGCTGCGGCGGAGAGCTCGCCGgactaCCGCCCGGAGATTGGTTGCTTGCGCTG |

# 2 Supplementary Sequence

>P*_OsUAH_*

TGACTTAGTAATGATAACAACACAGTACTTATTAGGAGCATTACAGCACCATAGTCACTAACCAAGATGTTCTTAAACTTCTAATAAAACTGCATCCCCTTCTCAGGGAGTAAAGAAAAGCAAGCAAGCCTACTCAAGCACTTGGTCACAAGTTAGAGCAGCATTAATTTGAAACTATTATTGAAATGGCATCATATTATATAATGTAAAAATGCCAGCTAGTTCCTCTTTCATTTCTGTCTCCTGTGTCAAAACACAATTTATTTTCAACTCCATTACTTGCAGACAACAGGCTCACCAACTATCAGATAGATTGGTAAAAGAAAATACAACAGTTTATAGACCATCGCAGTTGAATGTGAATATAATACATGGTGTGGGGGATCAAGAAATTGCATAGTGCTGTAACCACCTGTAACTACAATGCTTCCTAGCTAAATTCATTCCTCAATACAACTATAAAATAGGACAAAAATGGCACCCATATATTACAAATATCTACTTGACTGGTTCTCTGAAACAAGCGTACCGTGCATTCAAAGCCTACAACACTACTGCGCATACTGACTGAAACAACAATAGTAGAAACTAGTCCAGCTTAAGAAGAGAGAAAAAACTTACTGCTCTATCTGGTACCCCTGGTGGTCCATCAAGCACCTTTATGCGAGCTCTGGACTCCTCACACATTTTCTTGATAAACTCCCCTTTTCGGCCAATGACAGCGCCCACTTTTTGGGCTGGAACAAGTATACGGAACACACTTTCTCCAGGCCAACCTGGCCATTTCTTCTCATCAGTTACTGCATTGGCCTGCTGCTCTCCCTGAGCTTCTCCACCATAGGCATGCCCTTCTTCTGAGGGGATAGCTTGGTCCTCTTCATGCTGATGCCCCACATTTCCATCATTTATGTTCACCTGATCTTCATTGTATGGGATCCCTGATTCTTCATAGCTTGAGTCTTTGGTTTCTTCGTCATATGTATTTTCTGGATCTTCCTTGTACTGCTCAGGCACATCACTGAGAGGAATCGCCTGCTCCT
CATTATCAAGATTACTGGCAATCCCACTCATGTCCTCCGCAGGATTCTCGATGTTCCCATCCATATTTTCTGGAAACACACAACATAGACCATGTCAAGTAACACAAAACTGAAGGTGAGATGCTTCTCGGGCAGAAGTGCAGCATAAATTGTAGCAACAGGTGGCTCGCGTAATGCTTTGCGAGGGGCACAGCTCGCACGGAACAATACCAGATGGGGGCAACCCGCTTCCTGCACCAATCTAACTGTGGACTACACGAAAACCCCCAAGTTATTTCTACTCCGGATCCACCTAGATAAAGCTCAGCGCAACGCACAAACCCCTAACACCACAGCAAACTCGTCATAAGCGAGTGAAAAGAAAAAATCAGCCACGAGCCCCATAAACCCTAAATCGAGAGCAGAGCCGCGCCCCCACCACGACGCGAGCGGGCGCATCCAAGCTACACCTCCCGCGGCTACAGCGCGAGTACCACACGGCACAGGAGAACGAGGCCTACAGCGCAAGCAACCAATCTCCGGGCGGGTCGGCGGCGAGCTCTCCGCCGCAGCTACGCTAGACCCGCCCGCACCACCAAAGCTACGGATTCACCGGGAAGTAGATGCCGAGCGAAAGCGCGCTCACCTCACGGAGGAAGAGGACGAGGCCTCGCCAGAGAGGGAGTCCCGCCGCCGCCGCCGTGGAGGGTTTTGAGAGGAGAGGAGAGGAGAGCACGGGAGCCGTGCGGATTTGGGAGGAAAGAGAGAGAGAGAGTGTGTGGCTTGGTTGCCTCCTCGGTTTGATTTAACCTGGCTACTCCCACCAGCCCATGGGCCTCACGTTCGCCCACTCCTGGGCCCAGCCCATCCATATACCGGCCCACCGCTGCCAGGTGGGCCTCACTCCCCACCGTGCGCGGCCGCGGGCCCCACCGAAGAAACACCACACTCCCGTGACTCGTGTCGTCGTCTTCCTCGTCAGCCGCCACTGCCGTCGCGTCGTCTCCAACCTCCTCGCCTTCGCCTCCACCCAGCCATGGCGACGAGCGCCGCCGCGCGCT
TCCTCGCTGCCCTCGCGGGC
