# Supplementary figures and images for: Low-Temperature-Induced Expression of Rice Ureidoglycolate Amidohydrolase is Mediated by a C-Repeat/Dehydration-Responsive Element that Specifically Interacts with Rice C-Repeat-Binding Factor 3
Source: Front Plant Sci. 2015 Nov 13;6:1011. doi: 10.3389/fpls.2015.01011 (PMC4643140; doi:10.3389/fpls.2015.01011)

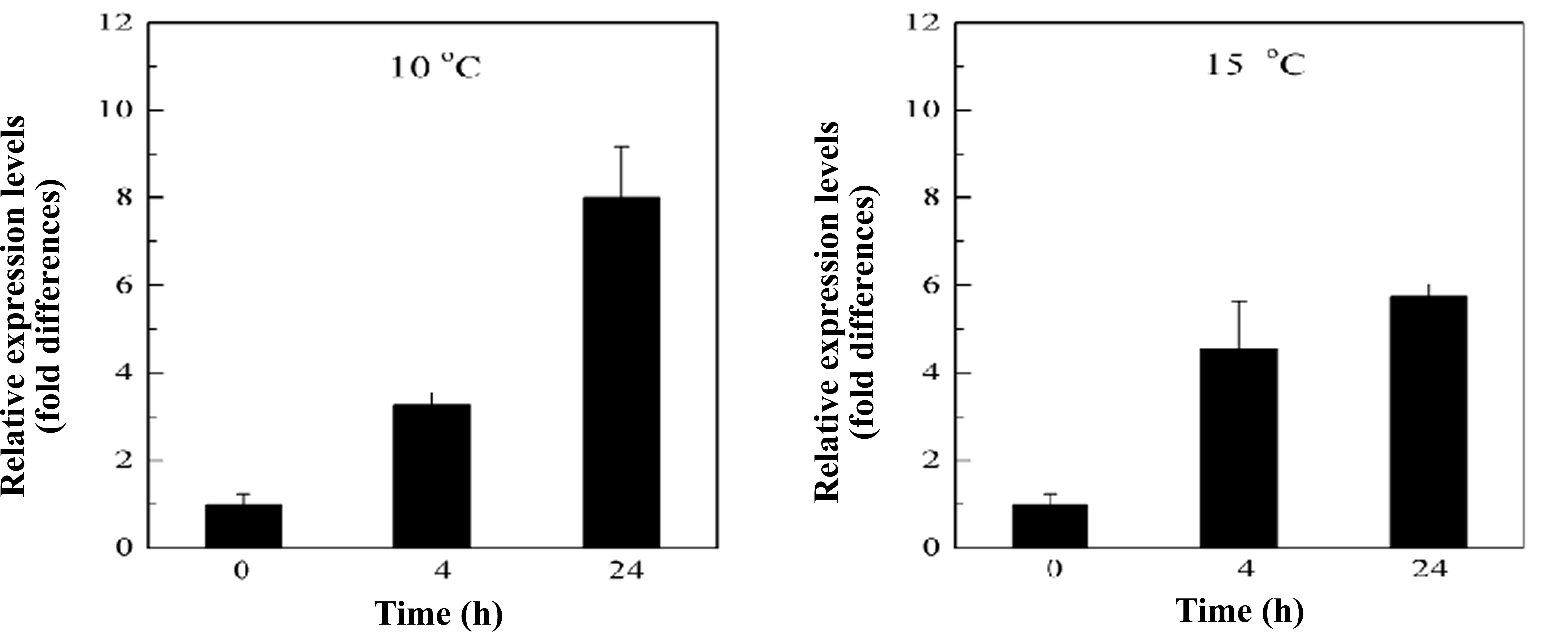

Supplement: Supplementary file 2 [file Image_1.TIF]

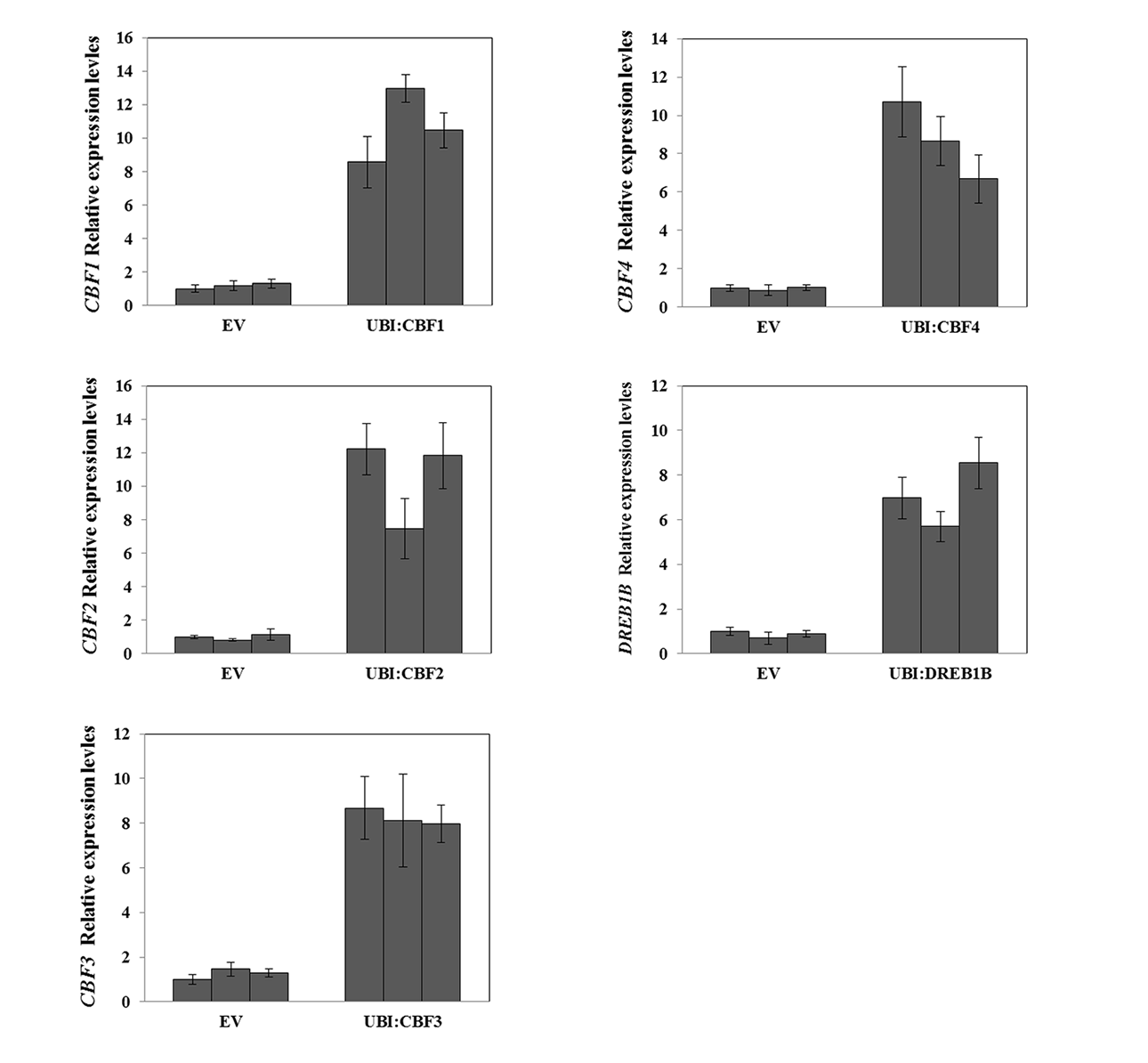

Supplement: Supplementary file 3 [file Image_2.TIF]

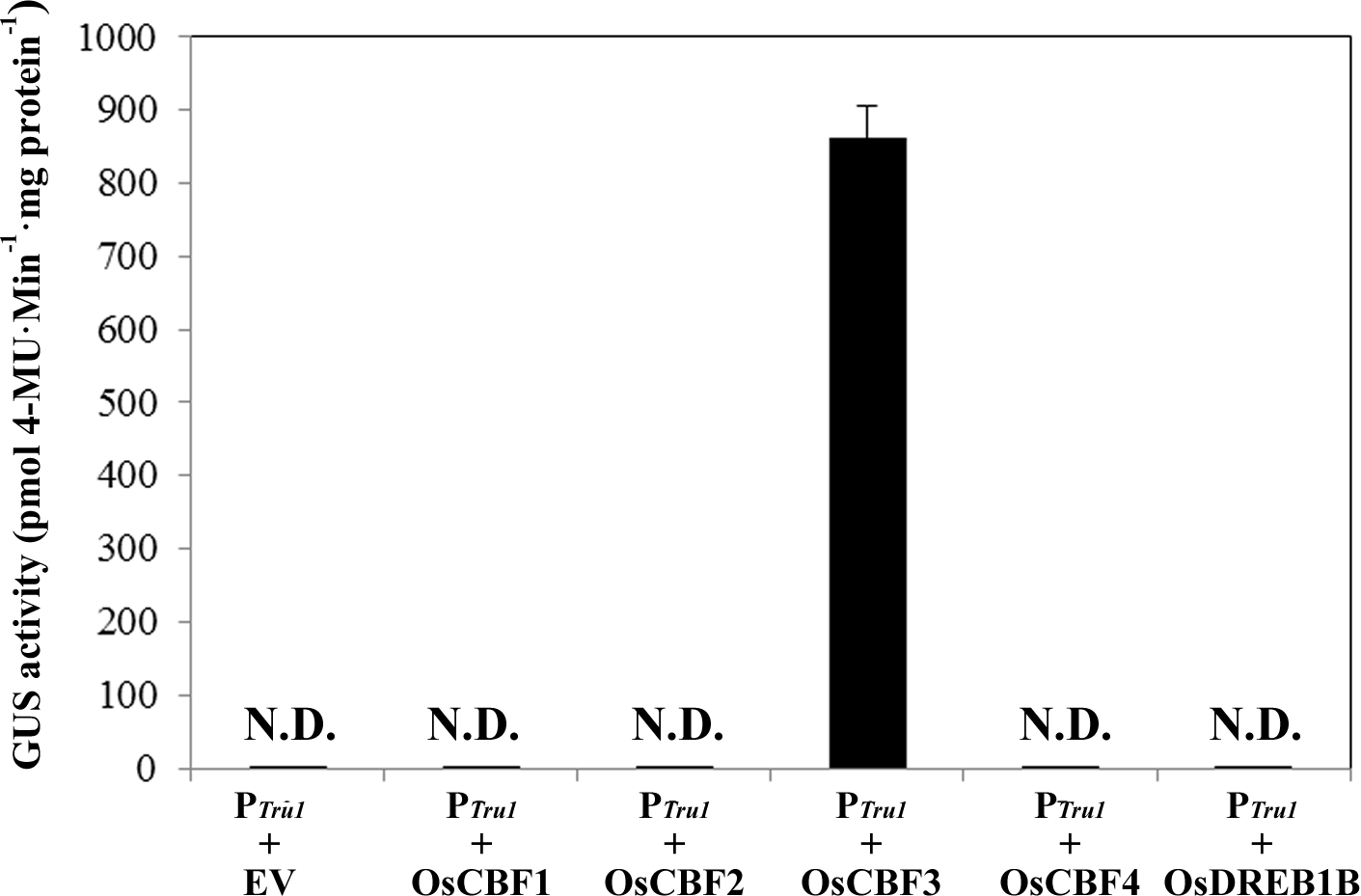

Supplement: Supplementary file 4 [file Image_3.TIF]
